# A case of *Bartonella* neuroretinitis with macular star diagnosed by clinical, epidemiological, serological, and molecular data: resolution after initiation of antimicrobial therapy

**DOI:** 10.1590/0037-8682-0516-2019

**Published:** 2020-06-22

**Authors:** Walter de Araujo Eyer-Silva, Letícia Stéfanie Curvello Wutke, Alexandre de Carvalho Mendes Paiva, Guilherme Almeida Rosa da Silva, Fernando Raphael de Almeida Ferry, Dario José Hart Pontes Signorini, Jonathan Gonçalves de Oliveira, Elba Regina Sampaio Lemos

**Affiliations:** 1 Universidade Federal do Estado do Rio de Janeiro, Hospital Universitário Gaffrée e Guinle, Centro de Ciências Biológicas e da Saúde, Rio de Janeiro, RJ, Brasil.; 2 Fundação Oswaldo Cruz, Instituto Oswaldo Cruz, Laboratório de Hantaviroses e Rickettsioses, Rio de Janeiro, RJ, Brasil.

**Keywords:** Bartonella henselae, Cat scratch disease, Ocular bartonellosis

## Abstract

The differential diagnosis of optic neuritis is broad and varied. We report the case of a 24-year-old Brazilian man who presented with five-week history of fever, malaise, myalgia, severe fatigue, tender right preauricular lymphadenopathy, and acute vision blurring associated with right optic disc swelling and exudates in a macular star pattern. His illness developed soon after an infestation of fleas broke out among his cats. Diagnosis of ocular bartonellosis was confirmed by serological and molecular analyses targeting amplification of *Bartonella* spp. *htr*A gene. Signs and symptoms only improved after initiation of antimicrobial therapy.

## INTRODUCTION

The eye is the most commonly affected non-lymphatic site in patients with cat scratch disease (CSD), a zoonosis caused by *Bartonella henselae* ^1^. The first clinical syndrome of what is now known as ocular bartonellosis was described in 1889 by a French neuro-ophthalmologist, Henri Parinaud, in patients with granulomatous follicular conjunctivitis, chronic fever, regional lymphadenopathy, and previous contacts with pets^1^. This syndrome is now known as Parinaud oculoglandular syndrome and ocular bartonellosis is one of its causes. Another clinical syndrome of ocular bartonellosis is neuroretinitis, a form of optic neuropathy characterized by optic disk swelling and eventual development of a partial or complete macular star. It was first described by a German ophthalmologist, Theodor Leber, in 1916^1^. The association of “Leber’s idiopathic stellate neuroretinitis” with CSD was first suggested by Sweeney and Drance in 1970^2^.

Herein we report the case of a Brazilian man who developed acute blurring of vision associated with optic disc swelling and exudates in a macular star pattern soon after an infestation of fleas broke out among his cats. Signs and symptoms remitted only after initiation of antimicrobial therapy.

## CASE REPORT

**FIGURE 1:**
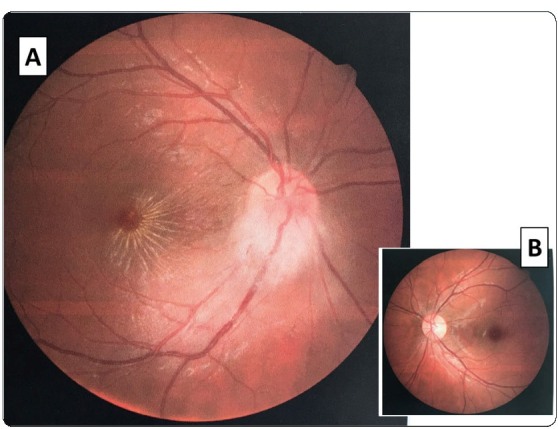
(A): a retinography image from a 24-year-old male patient shows a pattern of neuroretinitis with unilateral right optic disc swelling in the inferior margin and hard exudates in classic stellate distribution (macular star pattern); (B): The left eye was normal.

In late October 2018, a 24-year-old previously healthy male patient from the city of Rio de Janeiro, Brazil, developed a tender right preauricular lymphadenopathy and malaise. In early November 2018, he developed fever, myalgia, headaches, severe fatigue, and enhanced preauricular lymphadenopathy. On November 4, 2018, he suddenly noted blurring of vision in his right eye. There was no pain on ocular movement. He sought medical advice at another facility and was diagnosed with a “probable parotitis.” An otolaryngologist ruled out parotitis and prescribed a 7-day course of prednisone and a 14-day course of amoxicillin clavulanate for “adenitis”, leading to no clinical improvement. An ophthalmologist ordered a retinography that was performed on November 12, 2018. There was a pattern of neuroretinitis in the right eye, with optic disc swelling mainly in the inferior rim, and exudates in a macular star pattern (Figure 1). The left eye was normal. There was no conjunctivitis or ocular pain.

The patient first came to our attention on December 12, 2018. He still had blurred vision in the right eye, fever, malaise, fatigue, myalgia, as well as diarrhea. Visual acuity in the right eye was reduced to finger counting. Results of neurological examination were otherwise unremarkable. There was no serological evidence of syphilis, toxoplasmosis, viral hepatitis, or HIV infection. The clinical picture was considered consistent with neuroretinitis secondary to *B. henselae*. On detailed enquiry of his history, the patient informed that he owned three cats. He did not recall having been scratched or bitten by them. However, he did recall that in early October 2018, an infestation of fleas broke out and went out of control. The infestation was eradicated upon taking several flea control measures. 

Blood collected on December 13, 2018 was sent for serological and molecular analyses at a reference research laboratory with expertise in the diagnosis of bartonellosis. An indirect immunofluorescence assay for anti-*Bartonella* spp. IgG antibodies was positive at a titer of 1/512. Polymerase chain reaction (PCR) targeting the amplification of *Bartonella* spp. *htr*A gene, performed as previously described^3^, was positive. Five weeks later, the anti- *Bartonella* spp. IgG titer was 1/2048. Therefore, a diagnosis of *Bartonella* neuroretinitis was confirmed based on clinical, epidemiological, serological, and molecular data. The patient was treated with doxycycline (100 mg bid) for 14 days. He completely recovered and his vision improved rapidly, returning to normal (20/20). A retinal examination, two and six months after the completion of treatment showed complete resolution of the optic disc swelling, macular edema, and exudates. 

## DISCUSSION

The genus *Bartonella* is composed of facultative, intracellular, fastidious, aerobic, and oxidase-negative bacilli of the α2 subclass of Proteobacteria. More than 30 *Bartonella* spp. have been isolated from humans as well as from wild and domestic animals^4^. The genus is named after a Peruvian (Argentina-born) microbiologist Alberto Leonardo Barton, who described these bacteria in 1909 while studying the causative agent of Oroya fever. Common features of *Bartonella* species include transmission by an arthropod vector and survival within mammalian hosts that act as reservoirs.

*B. henselae* was first detected in 1990 through sequencing of 16S rRNA gene extracted from lesions of bacillary angiomatosis^5^, an infectious disease that causes proliferation of small blood vessels in the skin and visceral organs of HIV-infected and other immunocompromised patients. It was initially named *Rochalimaea henselae*, but was reclassified within the *Bartonella* genus as *B. henselae* in 1993^6^. Cats are the primary reservoirs for *B. henselae* and the infection is transmitted via the scratches of infected animals or by bites of the cat flea (*Ctenocephalides felis*). A paleomicrobiological study has reported evidence of *B. henselae* in the dental pulp of three cats dated from the 13^th^, 14^th^, and 16^th^ centuries found at different burial sites in France^7^, thus demonstrating its long-lasting association with a human pet. 

*B. henselae* is the etiologic agent of CSD, a common cause of subacute regional lymphadenitis. This zoonosis is usually described as a benign, self-limited illness. One complication of CSD is Parinaud oculoglandular syndrome, which presents with fever, regional lymphadenopathy, and granulomatous follicular conjunctivitis. Other complications include neuroretinitis, focal retinochoroiditis, iridocyclitis, papillitis, peripapillary angiomatosis, serous retinal detachment, branch retinal arteriolar occlusion, endophthalmitis, myelitis, aseptic meningitis, and encephalitis^8^ ^,^ ^9^. *B. henselae* is also one of the etiologic agents of bacillary angiomatosis (the other being *B. quintana*, a human-specific pathogen with worldwide distribution transmitted by the human body louse *Pediculus humanus humanus*). *B. henselae* is also an agent of culture-negative endocarditis, bacteremia, osteomyelitis, and bacillary peliosis.

The diagnosis of ocular bartonellosis in our patient was based on clinical, epidemiological, serological, and molecular data. Even though the blood sample could not be collected in the first days of his illness, a four-fold increase in anti-*Bartonella* spp. titer was demonstrated in samples collected five weeks apart. Additionally, PCR assay from blood samples resulted positive for *Bartonella* spp. *htr*A gene, thereby suggesting ongoing active disease six weeks after the start of clinical illness. Unfortunately, sequencing of the PCR product and flea vector sampling for microbiological analyses could not be performed, which could have provided further information on the molecular epidemiology of the present case.

A major feature of our patient’s illness was myalgia and fatigue. Musculoskeletal manifestations are generally considered rare in CSD. However, a large-scale Israeli study found that myalgia occurred in 53 (5.8%) of 913 CSD patients and was found to be a clinically significant manifestation of CSD, often with an unusually severe and protracted course^10^. Additionally, musculoskeletal manifestations such as myalgia, arthralgia, and arthritis occurred primarily in adults, and were occasionally prolonged, with some patients developing chronic disease. When we first saw our patient, he was complaining of myalgia, malaise, fever, and blurred vision for more than five weeks, and antimicrobial therapy had not been prescribed. A rapid remission was recorded once doxycycline treatment was initiated. 

Although there is no consensus on the utility of antibiotic therapy in the course of typical CSD, retrospective and prospective studies have found that the mean duration of illness was significantly shorter when patients were treated with certain antimicrobials such as rifampin, gentamicin, and azithromycin^11^. For patients with neuroretinitis, treatment should indeed be offered^4^, which generally is a combination of doxycycline and rifampin. A retrospective case series of *B. henselae* neuroretinitis found evidence that treatment with doxycycline and rifampin shortens the course of illness and hastens visual recovery^12^. In the present case, we offered doxycycline monotherapy resulting in excellent response.

The differential diagnosis of optic neuritis includes idiopathic inflammatory demyelination of the optic nerve, ischemic, and infectious disorders. Our patient was seen by several health care professionals, including a clinical ophthalmologist and a retina specialist, and yet the possibility of ocular bartonellosis was not raised. He remained symptomatic for several weeks before an appropriate antimicrobial agent was finally prescribed. It is of utmost importance that physicians keep a heightened awareness of the clinical bartonellosis syndromes. For instance, asking patients about recent history of cat contact is essential. A combination of clinical, epidemiological, serological, and molecular data may lead to the correct diagnosis. Rapid institution of treatment allows a shorter duration of illness and prevents permanent sequelae.
